# An Appointment Book

**Published:** 1891-12

**Authors:** 


### An Appointment Book.

PEARSON'S DENTISTS' APPOINTMENT BOOK FOR THE VEST POCKET, FOR 1892.

This little Annual, which has just been issued, is in a very convenient form for daily use in making appointments and keeping memoranda; it is the smallest Engagement Book issued, and on this account very convenient; it should be in the hands of every practicing dentist. It can be obtained by addressing, R. I. Pearson & Co., Kansas City, Mo.

